# Next generation media monitoring: Global coverage of electronic nicotine delivery systems (electronic cigarettes) on Bing, Google and Twitter, 2013-2018

**DOI:** 10.1371/journal.pone.0205822

**Published:** 2018-11-02

**Authors:** John W. Ayers, Mark Dredze, Eric C. Leas, Theodore L. Caputi, Jon-Patrick Allem, Joanna E. Cohen

**Affiliations:** 1 University of California San Diego School of Medicine, La Jolla, California, United States of America; 2 Department of Computer Science, Johns Hopkins University, Baltimore, Maryland, United States of America; 3 Stanford Prevention Research Center, Stanford University School of Medicine, Palo Alto, California, United States of America; 4 Keck School of Medicine, University of Southern California, Los Angeles, California, United States of America; 5 Bloomberg School of Public Health, Johns Hopkins University, Baltimore, Maryland, United States of America; Universidade Federal de Sao Paulo, BRAZIL

## Abstract

News media monitoring is an important scientific tool. By treating news reporters as data collectors and their reports as qualitative accounts of a fast changing public health landscape, researchers can glean many valuable insights. Yet, there have been surprisingly few innovations in public health media monitoring, with nearly all studies relying on labor-intensive content analyses limited to a small number of media reports. We propose to advance this subfield by using scalable machine learning. In potentially the largest contemporary public health media monitoring study to date, we systematically characterize global news reports surrounding electronic cigarettes or electronic nicotine delivery systems (ENDS) using natural language processing techniques. News reports including ENDS terms (e.g., “electronic cigarettes”) from over 100,000 sources (all sources archived on Google News or Bing News, as well as all news articles shared on Twitter) were monitored for 1 January 2013 through 31 July 2018. The geographic and subject (e.g., prevalence, bans, quitting, warnings, marketing, prices, age, flavor and industry) foci of news articles, their popularity among readers who share news on social media, and the sentiment behind news articles were assessed algorithmically. Globally there were 86,872 ENDS news reports with coverage increasing from 8 (standard deviation [SD] = 8) stories per day in 2013 to 75 (SD = 56) stories per day during 2018. The focus of ENDS news spanned 148 nations, with the plurality focusing on the United States (34% of all news). Potentially overlooked hotspots of ENDS media activity included China, Egypt, Russia, Ukraine, and Paraguay. The most common subject was warnings about ENDS (18%), followed by bans on using ENDS (13%) and ENDS prices (9%). Flavor and age restrictions were the least covered news subjects (~1% each). Among different subject foci, reports on quitting cigarettes using ENDS had the highest probability of scoring in the top three deciles of popularity rankings. Moreover, ENDS news on quitting and prices had a more positive sentiment on average than news with other subject foci. Public health leaders can use these trends to stay abreast of how ENDS are portrayed in the media, and potentially how the public perceives ENDS. Because our analytical strategies are updated in near real time, we aim to make media monitoring part of standard practice to support evidence-based tobacco control in the future.

## Introduction

Despite substantial popular interest in electronic nicotine delivery systems (ENDS), or electronic cigarettes [1,2], and research showing media plays a crucial role in guiding this interest [3], there is surprisingly little known about how ENDS are covered in the news [4,5]. This knowledge gap is likely a reflection of the substantial barriers to content analyzing news media. Data collection alone presents a host of challenges: What sources can be accessed? How should sources be searched to systematically identify relevant news reports (including non-English sources)? Among news reports, which should be selected for in-depth analysis? These challenges are amplified by similar hurdles in conducting the analysis itself: How can any researcher read hundreds of thousands of news reports? For example, one of the most ambitious studies to date analyzed 119 ENDS focused news reports covering 12 news outlets from 2 countries, finding ENDS coverage increased over the study period and news reports focused on how ENDS are used to avoid clean indoor law provisions, their health benefits, their price, and celebrities who use them [6].

Yet, news media monitoring is an important public health tool. By treating news reporters as data collectors and their reports as qualitative accounts of a fast changing public health landscape, researchers can glean many valuable insights [7]. Sickweather (www.sickweather.com), for instance, crowdsources media reports mentioning infectious diseases as proxies for potential outbreaks. News media monitoring has also been used to better understand policy debates related to addictive substances, such as the Scottish alcohol minimum unit pricing policy [8–9]. Given news is both a marker for ENDS’ rise and a conduit for how information (and misinformation) on ENDS spreads, news media monitoring can play a more important role. Where are ENDS garnering coverage? What ENDS issues are emerging? What regulatory strategies are being prioritized? These questions, and many more, can be answered by news media monitoring.

Herein, we demonstrate the feasibility of a machine led protocol to search, aggregate, and analyze media in a retrospective study of ENDS related news. The aim of this study is to systematically characterize global news reports surrounding ENDS using state-of-the-art machine learning and natural language processing techniques. Machine led protocols make it potentially viable to survey the entire universe of news reports surrounding ENDS and enable researchers to track where, how, and how much ENDS are being covered in different regions of the world. This protocol lays the groundwork for new opportunities in ENDS surveillance such as identifying populations where ENDS may be popular, understanding which stakeholders will be intertwined with ENDS regulation, and discovering the appeal or perceptions of ENDS by the public. As a result, this practice can cost-effectively help inform tobacco control priorities and opportunities in real-time.

## Materials and methods

In the first stage of this study, we developed a corpus of news sources which included all news reports archived on Bing News and Google News—the two leading search engine providers—including all available country specific domains and all URLs shared on Twitter. This provided monitoring of more than 100,000 news outlets in 23 languages worldwide, representing the largest and most diverse pool of media collected in tobacco control to date. This corpus includes both objective and subjective (e.g., editorials and op-eds) news articles, from mainstream or industry media outlets and traditional hard news (e.g., the New York Times) or soft news (i.e., blogs) sources.

In the second stage, we extracted ENDS related news from the corpus by searching for news reports using keywords and subsequently identifying the subset that were ENDS related. We queried our corpus to gather news reports published between January 1, 2013 and July 31, 2018 that included the following ENDS-related keywords or their Arabic, Chinese, Indonesian, Portuguese, Russian, Spanish and Vietnamese translations: electronic cigarette(s), e cig(s), e-cig(s), ecig(s), e cigarette(s), e-cigarette(s), ecigarette(s), vape(s), vaper(s), and vaping. (Our selection of languages was guided by the Bloomberg Philanthropies’ tobacco control priorities and our internal capabilities.) These queries yielded 105,550 matching news reports. We then identified the subset of reports primarily focused on ENDS. By relying on a combination of the number of product mentions and the order of product mentions, we developed a scoring system to automatically estimate whether each article was primarily ENDS focused. We used machine learning techniques to select the weighting of this formula against a training set of human annotated documents where readers identified the primary subject of a report qualitatively. The precision (or positive predicted value) of these automated classifications compared to human led annotation was 0.81, meaning the automated classifier correctly predicted the human annotators choices 81% of the time. This precision surpasses common standards (0.70) for natural language processing [10]. Applying this classifier to the entire database resulted in 56,140 selected news reports for our analysis.

In the third stage, we aimed to contextualize our understanding of ENDS news by (a) discovering the geographic foci of ENDS coverage, (b) the subject theme of news reports, (c) what news reports are popular among the public, and (d) the sentiment that is conveyed in ENDS news.

### Geographic foci

Typically automated content analyst rely on proxies for geolocation, such as the address of the publishing source. However, in practice these strategies generate substantial errors. For example, the New York Times regularly publishes news about areas outside of New York. We relied on validated strategies [11] to cluster news reports according to their geographic-subject focus by incorporating the geographic distribution of named entities (both locations, such as “Moscow,” and objects, such as “Centers for Disease Control and Prevention”) in the text. If only one location entity was mentioned in a report, it would be given the corresponding national geographic-subject location. When more than one location entity was mentioned, the national geographic-subject location was determined by a majority of mentions. News reports with all entities receiving 50% or fewer mentions were labeled as multinational. This algorithm was selected to optimize accuracy, yielding 0.78 precision against human led annotation based on qualitative judgements.

### Subject foci

Subject element classification focuses on clustering reports according to major topical domains. Using the World Health Organization’s MPOWER policy package [12] as motivation, we compiled an initial listing of potential subject areas focused on product regulation. We then identified potential areas of poor or incomplete fit with the MPOWER package and created additional labels such as age restrictions. Through this method, we developed 9 subject categories: prevalence (e.g., estimates for ENDS use), bans (e.g., bans on using ENDS, such as indoors), quitting (e.g., using ENDS for cessation), warnings (e.g., the potential harms of ENDS use), marketing (e.g., ENDS marketing), prices (e.g. taxes or minimum price regulations for ENDS), age (e.g., restrictions on minimum age to purchase ENDS), flavor (e.g., ENDS flavors), and industry (e.g., industry activities). Except where obvious (e.g., age restrictions), these subject categories did not differentiate between youth and adult focus. A training set of articles (n = 5,000) was assessed and given a primary subject classification for each of the above labels on Amazon’s Mechanical Turk using a Turkle web-based crowdsourced annotation platform [13]. Two annotators labeled reports according to their subject. When the primary subject of the report did not match our subject labels or annotators disagreed (about 5%), the report was coded as “other.” We then developed a supervised machine learning classifier relying on support vector machines and recurrent neural networks [14,15], which reflect state of the art approaches in the deep learning community, to optimize automatically inferred subject prediction. The precision of these automated classifications compared to qualitative human lead annotation was 0.70.

### Popularity score (i.e., “trending score”)

Understanding the popularity of ENDS news reports can help distinguish between the priorities of reporters and consumers. Popularity was measured in two dimensions: pagerank and social media shares. The source for each news report was scored using Google PageRank for the source URL. PageRank represents how frequently a page is linked to other sites and is the benchmark for generating the order of results returned to a Google search. Each mention of a news report on Twitter and Facebook was monitored (via their respective Application Programming Interface (API)) and the number of shares recorded, including all shares pointing to the report’s URL. We used a studentized scale combining the two, giving equal weight to all sources of data, to describe any news report’s popularity.

### Sentiment score

We captured the prevailing sentiment of each news report to determine whether each report contained positive, negative, or neutral language using Google’s Natural Language API (https://cloud.google.com/natural-language/). Using a modified bag of words approach [14], each news report was broken into sentences and each sentence assigned a prevailing sentiment score. The resulting scores were then analyzed on a -1 to 1 scale with 0 indicating neutral sentiment, negative values negative sentiment, and positive values positive sentiment. Scores deviating by at least -0.5 or 0.5 are typically considered strongly negative or strongly positive, and that threshold was used here to label news reports as categorical negative, neutral or positive. For example, an article with the title, “Infant case of nicotine poisoning demonstrates harms of e-cigarettes”had a score of -0.7, whereas a company-written press release for an up-and-coming vape shop received a +0.5 score.

Using the above data and labels we performed an exploratory analysis of ENDS-related news. We plotted ENDS-related news volume over time, explored recurring patterns (e.g., day of the week [16–18]), and used a simple linear regression to estimate any linear time trend. We replicated this analysis by ENDS news reports’ location, subject, trending score and sentiment score, and results were described and compared against each other.

All analyses relied on anonymized data and adhere to the terms and conditions, terms of use, and privacy policies of Bing, Google, and Twitter. All analyses were computed using R Ver. 3.2.1.

## Results

**Fig 1:**
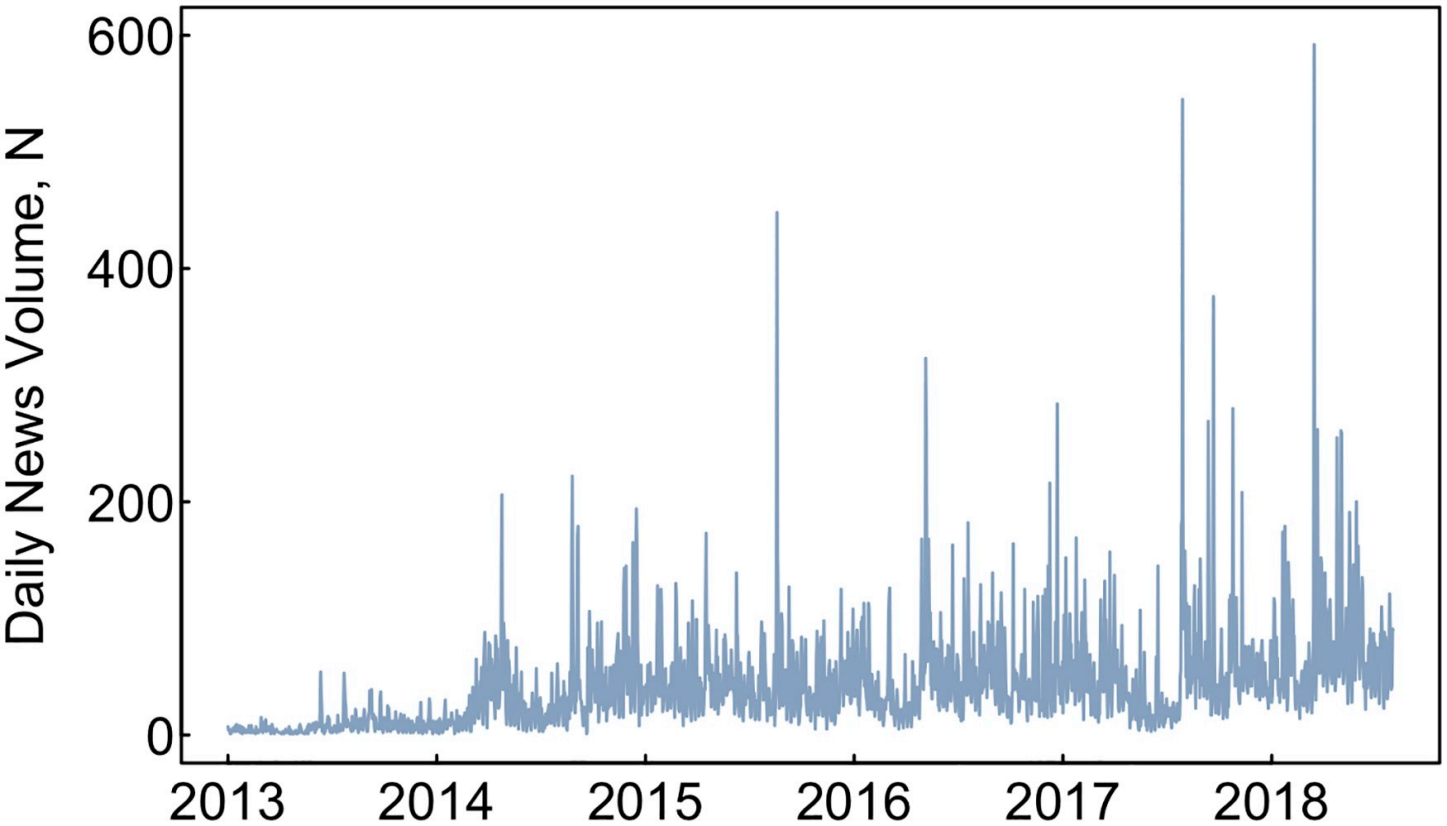
Daily ENDS news reports, 2013–2018. The above figure shows daily news volume trends for all news articles. This is a simple count of articles by day over the study’s timeframe.

Globally there were 86,872 primarily ENDS related news reports (in the 11 languages considered) from January 1, 2012 to July 31, 2018. The lowest day had no ENDS news reports, and the highest day had 592 reports on March 15, 2018 when stories about quitting smoking by using ENDS dominated the news (Fig 1). News volume trended upward, increasing from an average of 8 (standard deviation [SD] = 8) stories per day in 2013 to 36 (SD = 34) in 2014, 47 (SD = 34) in 2015, 51 (SD = 40) in 2016, 57 (SD = 33) 2017 and 75 (SD = 56) for the partial 2018 year. Apart from the upward trend there was no other distinct recurring patterns, such as seasonality.

### Geographic foci

**Fig 2:**
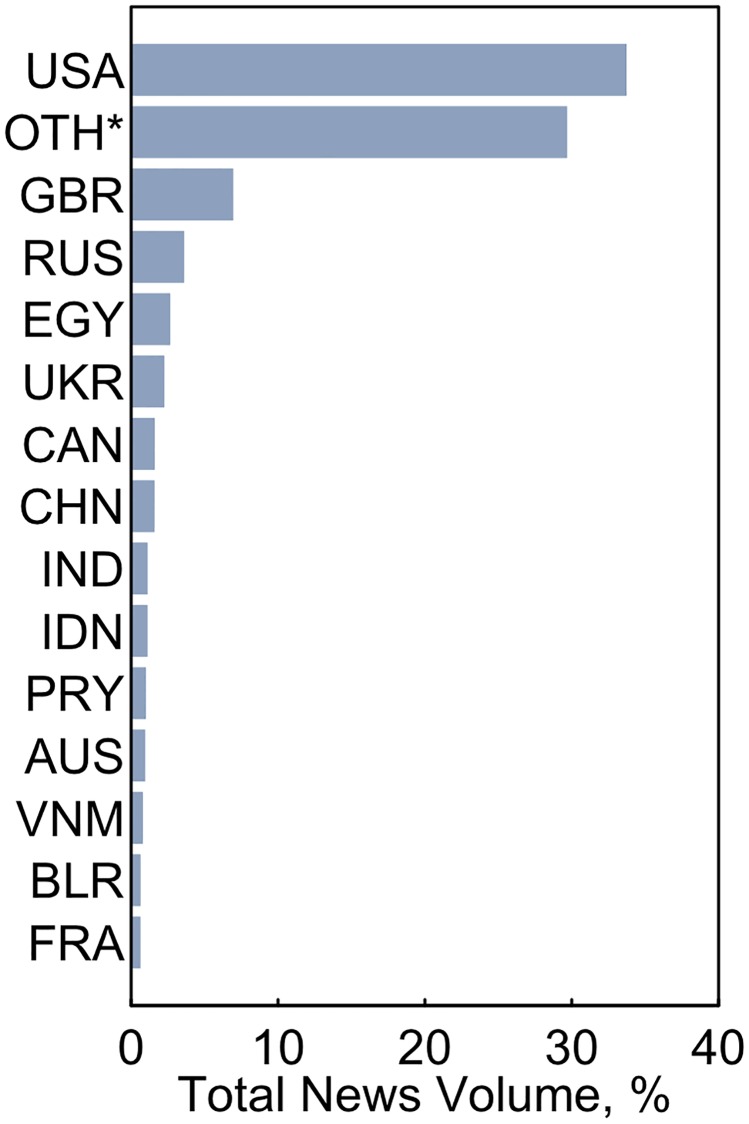
Proportion of all news reports by location, 2013–2018. Article geographic foci is discerned using a machine learning classifier that accounts for location-identifying words in the article and reports the location with the most mentions. Results are restricted to the 15 most covered geographies with “oth*” indicating a multi-national focus and other labels matching ISO 3166 Alpha-3 abbreviations.

The geographic foci of ENDS news reports altogether spanned 148 nations (Fig 2) with much of the news focused on the United States (34% of all reports) and Great Britain (7%). Together, the 14 most-cited nations together accounted for 59% of all ENDS focused news coverages. These top rankings revealed some potential previously overlooked hotspots of ENDS activity, including Egypt (3%), Russia (4%), and the Ukraine (2%), highlighting the global scale of ENDS’ reach. Other notable nations by region included Paraguay with the highest news volume in Latin America (~1%), China in East Asia (2%), India in West Asia (~1%), and Indonesia (~1%) and Vietnam (~1%) in the South Pacific. In addition to national news coverage, a substantial number of articles had multinational foci featuring, in similar detail, 2 or more nations (30%).

### Subject foci

**Fig 3:**
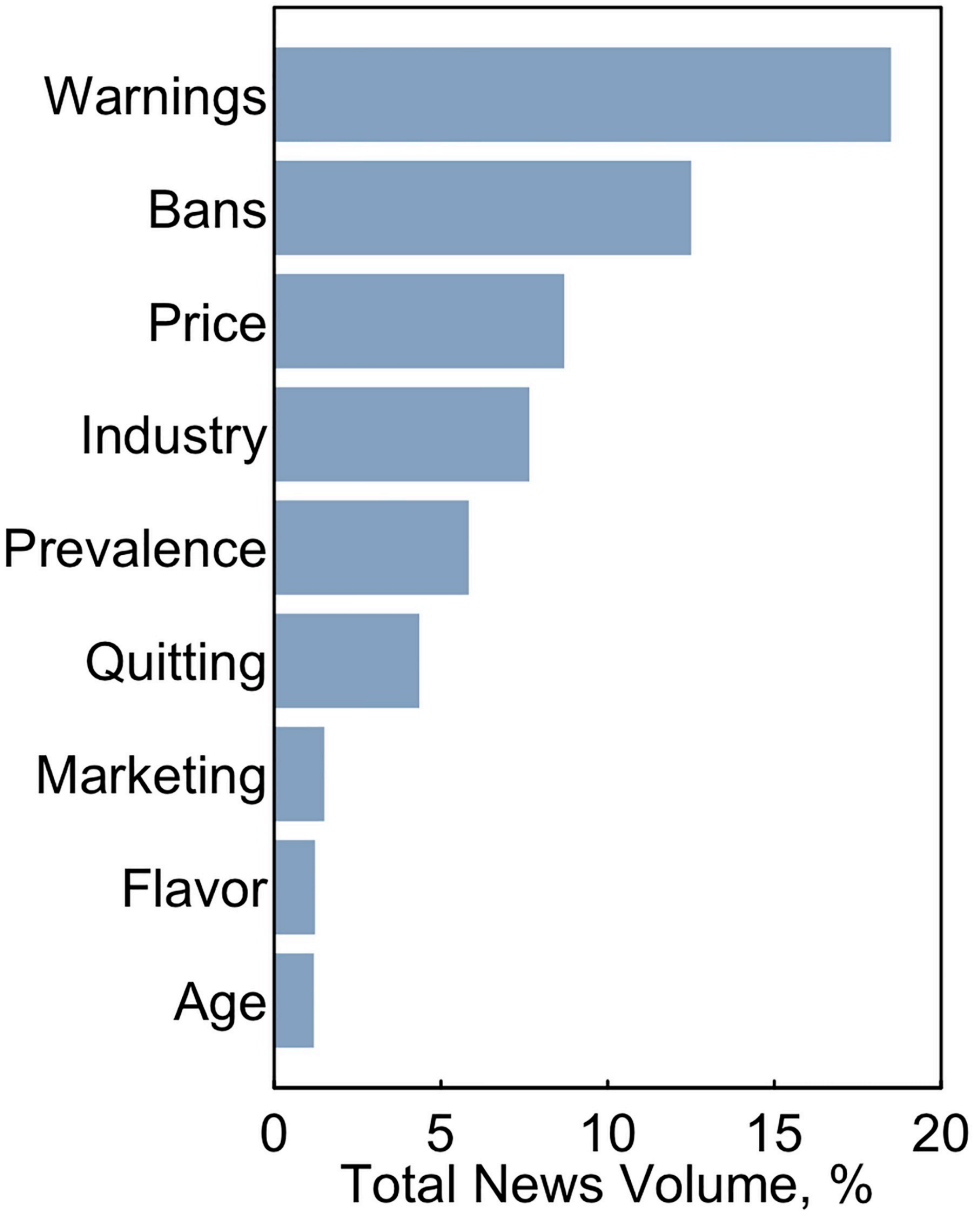
Proportion of all news reports by their subject focus, 2013–2018. Article subject foci is discerned through a supervised machine learning classifier relying on support vector machines and recurrent neural networks, which used a training set of 5,000 news articles coded using a Turkle-based application on Amazon Mechanical Turk. News reports without a subject foci matching our classifiers are not shown but represent 39% of all ENDS news reports.

Among all ENDS news reports the most common subject focus was warnings (Fig 3); news reports highlighting the health implications of using ENDS or warning against their use made up 18% of all news reports. News about warnings was followed in prevalence by news about ENDS bans (13%), ENDS prices (9%), the ENDS industry (8%) and prevalence of ENDS use (6%). Out of the topics we assessed, the least prevalent were the use of ENDS to quit smoking (4%), ENDS marketing (2%), flavor restrictions (~1%) and age restrictions (~1%).

**Fig 4:**
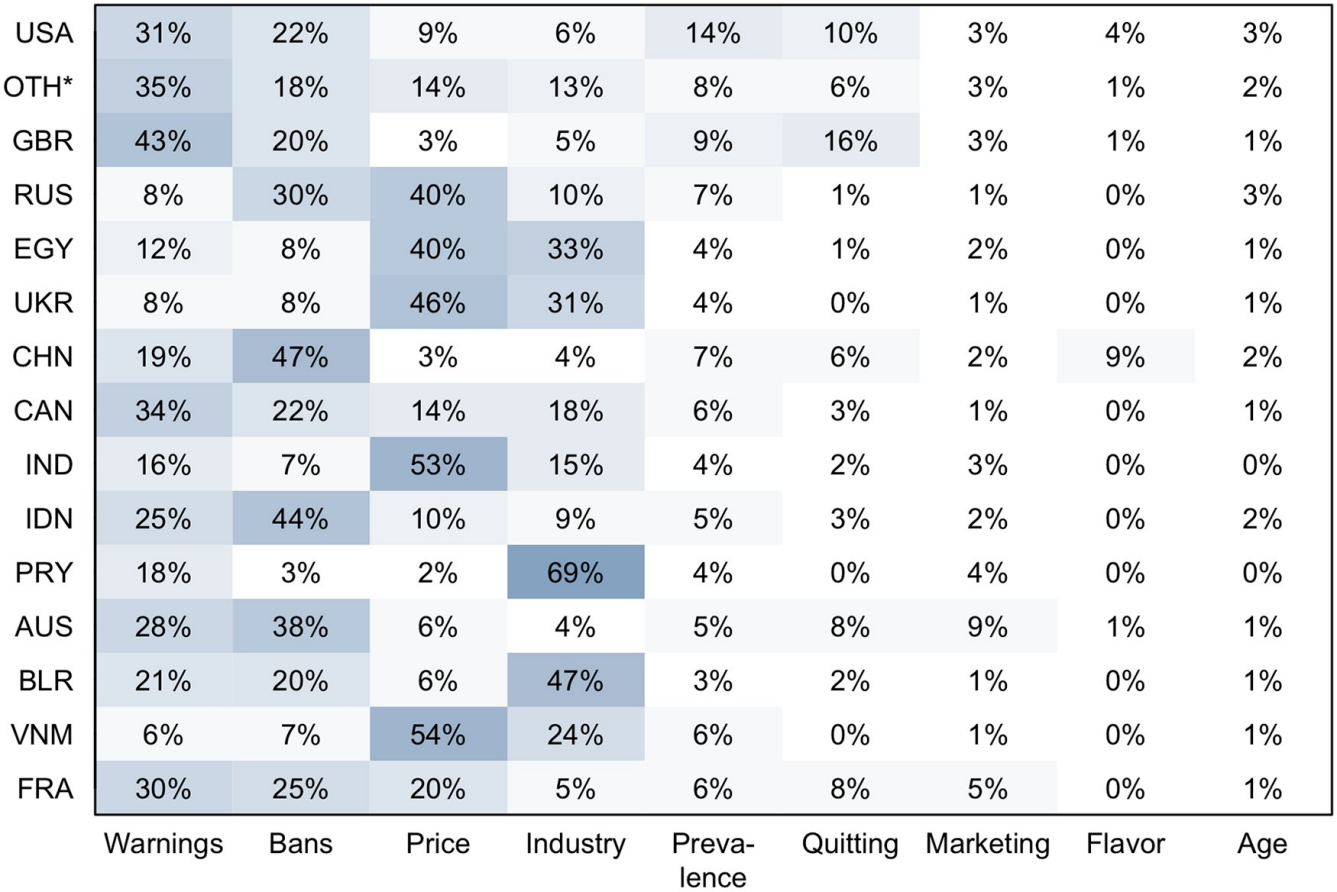
ENDS news subject foci by geography, 2013–2018. The above figure shows the crosstabulation of location and subject foci for ENDS news reports published between 1 January 2013 and 1 Aug 2018. Rows reflect the focal location, including all ENDS news and ENDS news for the 15 most covered locations using ISO 3166 Alpha-3 abbreviations, and columns reflect the focal subject for ENDS news reports. The numbers in each cell represent the percentage of all news coverage in each nation that was classified into each of the MPOWER categories. Darker shades represent higher percentages. Unlabelled news reports are not shown but include 39% of news reports. Article geographic foci is discerned using a machine learning classifier that accounts for location-identifying words in the article and reports the location with the most mentions. Article subject foci is discerned through a supervised machine learning classifier relying on support vector machines and recurrent neural networks, which used a training set of 5,000 news articles coded using Amazon Mechanical Turk.

There was variation in the prevalence of topics across countries (Fig 4). For example, 31% of ENDS-related news reports in the United States focused on warnings (making warnings the most common topic in the US) compared to just 8% of ENDS-related news reports in Russia. Additionally, nations like Egypt, Russia and Ukraine showed a high prevalence of news articles regarding ENDS’ pricing. In part, the variation of these may be indicative of ENDS-related issues facing each nation.

### Popularity score

**Fig 5:**
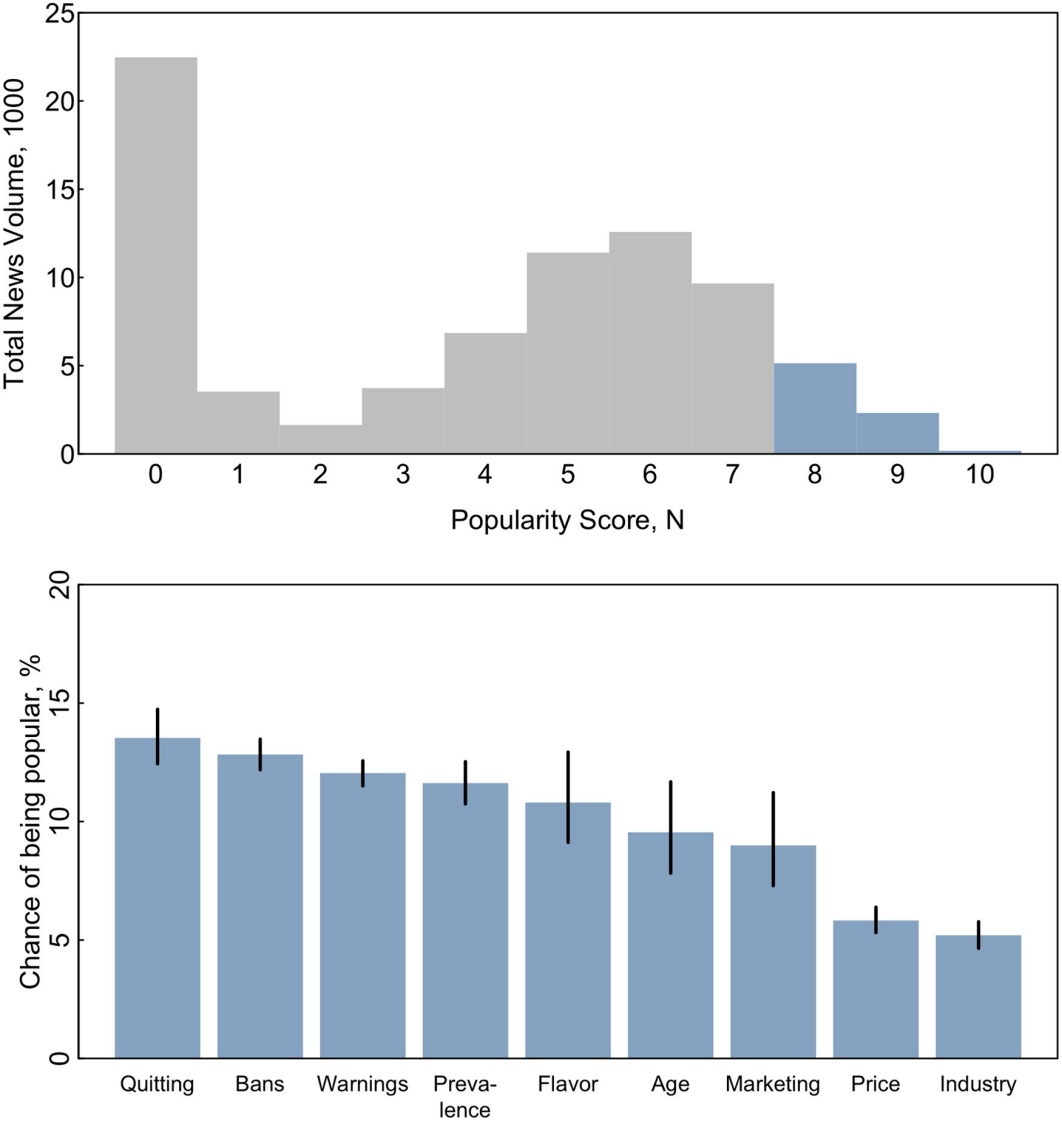
Popular ENDS news, 2013–2018. Panel A shows the distribution of popularity scores (representing their popularity online and via Google PageRank and shares on social media) for all ENDS news reports. Articles in the blue portion of the distribution were labeled “popular.” Panel B shows, for each subject foci, the chance of an article being popular (i.e., popularity score 8 or above) given only its subject is known, estimated from separate logistic regression models predicting popularity (yes/no) with dummy indicators subject categories. Popularity scores are calculated on a standardized scale that accounts for the reports’ Google PageRank score (indicating the number of webpages linked to it) and social media (Facebook and Twitter) shares.

Fig 5 shows the distribution of trending scores for all ENDS news reports. As expected, this distribution is right-skewed with few reports garnering substantial public attention and a high proportion of reports garnering little or no attention. Compared to less popular news, trending news reports typically included greater coverage of quitting, bans, and warnings. For example, there were few ENDS reports focused on quitting but these reports had the greatest chance of being popular, with an 13% (95%CI 12–15) probability of scoring in the top 3 deciles of popularity rankings. While the industry was the focus of nearly twice as many news reports as quitting, these articles had the least chance of scoring in the top 3 deciles of popularity rankings (5%; 95%CI 5–6).

### Sentiment score

**Fig 6:**
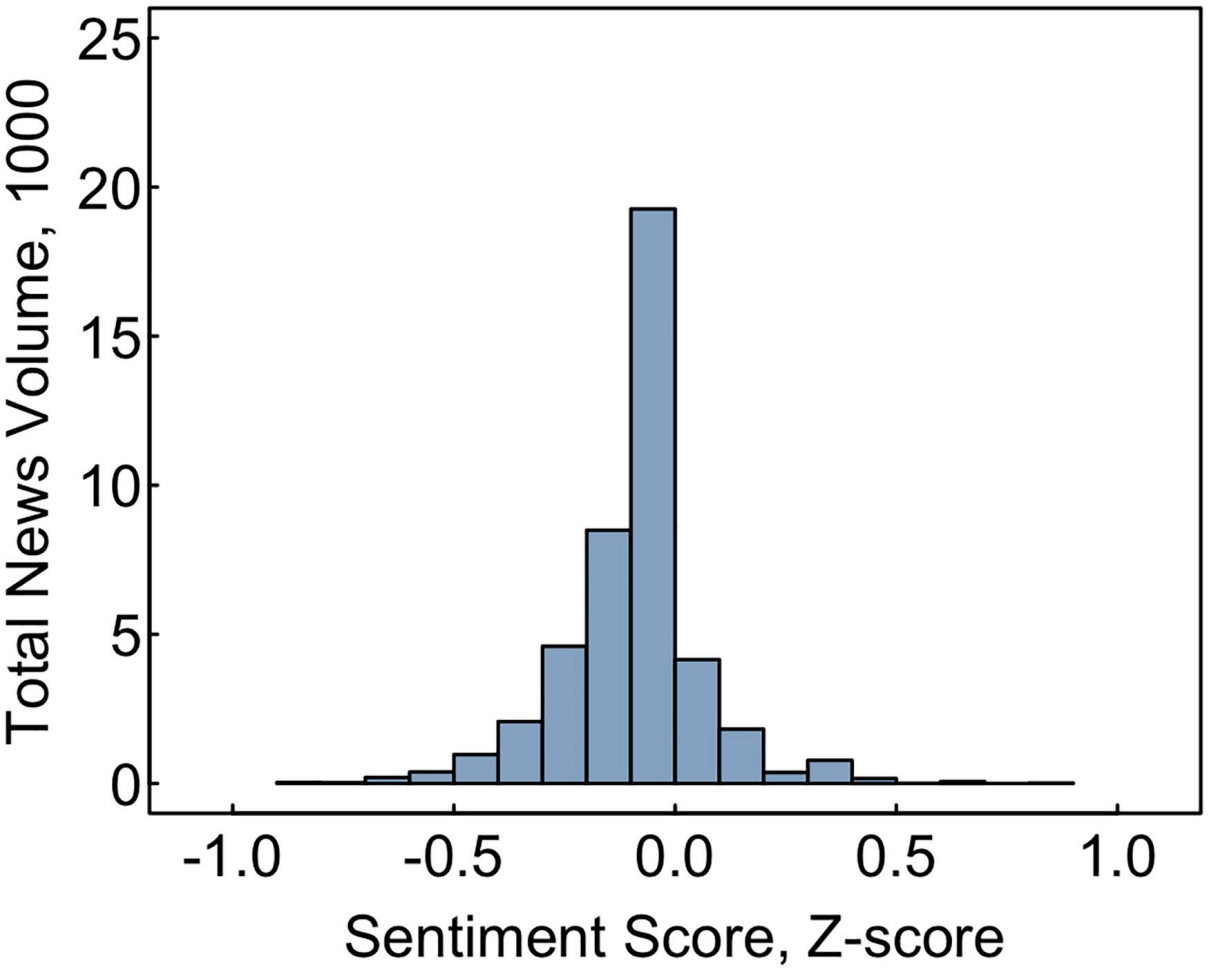
Distribution of ENDS news sentiment scores, 2013–2018. The above figure shows the distribution of sentiment among all ENDS news with 0 reflecting no sentiment, > 0.01 or < -0.01 indicating positive or sentiment and > 0.05 or < -0.05 indicating strongly positive or negative sentiment. Sentiment is calculated using Google’s Natural Language API and a modified bag-of-words approach, analyzing the distribution of negative, neutral, and positive words in the article.

Most ENDS news reports had neutral sentiment, with 44% of all articles having a sentiment score of approximately zero (Fig 6). However, the overall tendency was for a plurality of articles to have a negative sentiment (Mean = -0.042; SD = 0.16) with 2% of articles strongly negative (≤-0.05) and 37% somewhat negative (> -0.05 and < 0). Very few reports (17%) had positive sentiments (≥ 0.01) with just 509 (~1%) being strongly positive (≥ 0.05). Sentiment did not vary substantially by nation or trending score. However, news reports about quitting and prices were typically more positive that articles with other subject foci. For instance, 9% of reports on quitting were somewhat positive.

## Discussion

Our analysis of more than 85,000 ENDS-related news articles yielded several actionable insights. ENDS news reports are typically focused on the West (e.g., the United States and Great Britain) with some potential hotspots of overlooked activity (e.g., Egypt and Russia). Warnings were the most common subject focus. The most popular news articles tended to focus on quitting, bans, and warnings. While most news was neutral, articles typically had a slightly negative sentiment, except for those on quitting combustible tobacco which were written more positively.

The tools we developed have the potential to accelerate the frequency and scope of media monitoring, making it more useful for evidenced-based decision making. For example, tobacco control practitioners could use a real-time, automated system based upon these methods to capture which news topics are systemically important at a given moment in the discourse and then take appropriate action. Trends captured through our approach are likely more representative of the population of news sources and capture more articles, thereby providing a more valid and holistic understanding of the global news landscape in tobacco.

A bibliometric analysis found less than 9% of all research focused on understanding the broader tobacco landscape, even though tobacco control in practice aims to address and foster environmental shifts [19]. The paucity of research is more striking when only media monitoring is considered. A PubMed search for “news” and “content analysis” and “tobacco” returned just 34 reports at the time of the writing of this report. In contrast, advocates rely heavily on media monitoring to stay abreast of a changing landscape or monitor message replication in news reports. Moreover, the tobacco industry themselves rely on billions in market research to ensure messages that promote their products trend in the media.

Media monitoring in public health research is not only rare but, when available, it is on a small scale; studies typically monitor news in a single language, from a handful of sources, to analyze dozens of reports. Even so, much has been learned from media monitoring, including the tobacco industry’s role in independent military newspapers [20], popular perceptions of tobacco policies [21–24], and support for tobacco control in the news [25]. Moreover, several studies have relied on simple keyword-based media monitoring to inform tobacco control program evaluations and designs [26–29]. By making expansive media monitoring feasible (i.e., our study encompasses more news reports than the entire history of tobacco control media monitoring to date)the potential for even more impactful discoveries in the future is increased. This is especially true in a low information environment, where there are gaps in traditional data. For example, ENDS’s recency to market and regulatory ambiguities have made information sparse, and so novel or big data has been leveraged to develop some of the most central research in the field [2, 30–33], even predicting ENDS’ substantial growth as early as 2011 [1].

There are, of course, weaknesses to our approach. While largely automated media monitoring can capture a wide scope of media related to a given topic, it may lack the depth of human-led content analysis. Given the rapidity of changes within the tobacco control landscape, using these methods would potentially require adaptation to emerging topics and terminology (e.g., “Juuling” is a new, brand-specific term for “vaping”). However, researchers would become aware of this need far sooner using our system. Further, while the precision of our analysis, as determined by its correspondence with human-led coding, is high, our analysis is not as precise as experts reviewing individual news reports by hand. Additionally, while we identified the overall sentiment of news articles, a post classified as negative does not necessarily mean the news article was against ENDS. Rather, it meant the news article had more words conveying negative sentiment than positive [34]. Still these trade-offs weigh modestly against the potential benefits of our automated strategies.

Our findings highlight several disconnects between the scientific community’s priorities and the potential areas of concern among the public. There has been almost no published research on ENDS in Egypt, Russia, Ukraine, and Paraguay even though these are hotspots of ENDS news reporting and, potentially, areas of ENDS market growth. Moreover, while researchers, particularly in the United States, often cite flavor restrictions as a pressing area of ENDS regulations given that flavors are banned in other tobacco products, news reporters are not spreading this same message. Globally, flavor was the least cited focus area of ENDS news, ranking near the bottom for all of the top 15 countries, including the United States. Moreover, the most popular news among the public was on cessation, with cessation news also including more positive portrayals than other ENDS news. While work on ENDS as a cessation device remains limited and controversial in academia [35–37], the public appears eager to learn more and news writers often discuss cessation via ENDS positively. Work must begin now for the tobacco control community to more effectively get its priority messages propagated in news outlets and simultaneously to learn from news media monitoring how priorities must adapt.

News media monitoring can become part of the typical research process, serving as formative science [38] or early detection research, both for ENDS and across public health. Public health leaders can use these trends to stay abreast of the public’s perception of ENDS and how ENDS are portrayed in the media. Future analyses can be conducted on different, emerging topics in tobacco control, such as heated tobacco products [39–40]. Because our media monitoring strategies are updated in near real time, comprehensive, and automated, we aim to make them part of standard practice to support evidence based practice in the future.
